# The Effect of Alkaline Treatment on Poly(lactic acid)/Date Palm Wood Green Composites for Thermal Insulation

**DOI:** 10.3390/polym14061143

**Published:** 2022-03-12

**Authors:** Hyder Al Abdallah, Basim Abu-Jdayil, Muhammad Z. Iqbal

**Affiliations:** 1 Chemical and Petroleum Engineering Department, United Arab Emirates University, Al Ain 15551, United Arab Emirates; 201870175@uaeu.ac.ae (H.A.A.); mziqbal@uaeu.ac.ae (M.Z.I.); 2 National Water and Energy Center, United Arab Emirates University, Al Ain 15551, United Arab Emirates

**Keywords:** polylactic acid, date palm wood fibers, alkaline treatment, heat insulations, crystallinity, density, thermal conductivity

## Abstract

In this work, the effect of alkaline treatment on the thermal insulation and mechanical properties of date palm wood fibers (DPWF) and polylactic acid (PLA) green composite was studied. Alkaline treatment was applied to DPWF using two different solutions: sodium hydroxide (NaOH) and potassium hydroxide (KOH), with concentration of 2 vol.%. The fibers were later incorporated into PLA with weight percentages from 10 to 40 wt.%, to form three composite types: PLA with untreated fibers (PLA-UTDPWF), PLA with KOH treated fibers (PLA-KOH), and PLA with NaOH treated fibers (PLA-NaOH). The prepared composites were for use as a green thermal insulation material. The composites were tested to assess the effect of treatment on their physical (density and degree of crystallization), thermal (thermal conductivity, specific heat capacity, thermal diffusivity, thermal degradation, glass transition, and melting temperature), and mechanical properties. Moreover, the composite structural characteristics were investigated using FTIR and SEM analysis. The alkaline treatment significantly increased the crystallinity of the composites, specifically for higher filler loadings of 30 and 40 wt.%. The crystallinity for the 40 wt.% increased from 33.2% for PLA-UTDPWF, to 41% and 51%, for PLA-NaOH and PLA-KOH, respectively. Moreover, the alkaline treatment reduced the density and produced lighter composites than the untreated specimens. For instance, the density of 40 wt.% composite was reduced from 1.43, to 1.22 and 1.30 $\frac{g}{{\mathrm{cm}}^{3}}$ for PLA-NaOH and PLA-KOH, respectively.

## 1. Introduction

The primary source of carbon dioxide and greenhouse emissions is the high consumption of energy that is produced using fossil fuels. A large portion of the supplied energy is devoted to providing appropriate conditions within residential and commercial buildings. Buildings consume 40% of global energy production, which represents about 30% of carbon emissions worldwide [1]. In countries with extreme weather conditions, the amount of energy devoted to maintaining livable conditions within buildings is relatively higher. Furthermore, it is anticipated that the percentage of urbanization worldwide will increase to 73% by 2030, which will increase the demand for energy in the construction and building sector [2]. Heat insulators have an essential role in maintaining temperatures within buildings and reducing energy lose, leading to a reduction in carbon emissions. Commonly used heat insulators in buildings include polyurethane, polystyrene, mineral wool, aerogel blankets, and fiberglass [3]. Nonetheless, some of these materials pose a potential threat to health and the environment. Polystyrene is derived from petroleum, which makes its production environmentally damaging. Polyurethanes are synthesized from isocyanates, which are highly toxic for the environment and hazardous for health [4]. Moreover, glass wool and fiberglass are reported to cause respirational health concerns [5,6]. Therefore, producing green heat insulators that are based on biodegradable, sustainable, and ecological materials is imperative.

Bio-composite materials are a prominent solution with good potential for heat insulation. Numerous studies have been carried out about novel materials, to examine their capabilities for the purpose of heat insulation. Natural fibers such as flax, hemp, jute, pineapple leaves, sisal, seeds of coir, cotton, and palm are compounded with polymers to reinforce the matrix for construction purposes, including insulation [7]. For example, cork powder with 50 wt.% was used as reinforcement for polypropylene and polyethylene as fireboard materials [8]. However, several studies have investigated compounding natural filler into biopolymers, to form green biodegradable composites. Composites that are based on biodegradable polymers such as cellulose, starch, poly-lactic acid (PLA), poly-vinyl alcohol (PVOH), poly-butylene succinate (PBS), or poly-hydroxy butyrate (PBH) are being studied for various applications, including medical, agricultural, and packaging [9]. Among the mentioned polymers, PLA has received extensive attention over the years for its biocompatibility and stability, and it is being produced commercially on a large scale [10]. PLA matrices were reinforced with different filler materials, including natural fibers, in various recent research studies. Cellulose from agriculture waste (cotton) and industrial waste (paper) was incorporated into a PLA matrix, and the produced bio-composites were characterized [11]. In addition, wood, basalt fibers, ZnO, TiO_2_, jute, and flax were compounded with PLA, to study the effects of the reinforcement on the thermal, hydrothermal, and mechanical properties [12,13,14,15]. Overall, PLA possess excellent mechanical properties, relatively low thermal conductivity, and a relatively hydrophobic nature [16]. On the other hand, date palm wood fiber (DPWF) is an abundant natural fiber that is generated by date palm trees. DPWF is considered a huge agricultural waste, especially in countries where palm trees grow abundantly. Developing a composite material that consists of a PLA matrix reinforced with DPWF would be a good choice for making a biodegradable green heat insulation composite material. Meanwhile, using DPWFs as a filler provides a solution to the agricultural waste of date palm trees. Two previous works investigated the potential for PLA with date palm waste composites, to be used as heat insulators in construction. The two studies examined the incorporation of date pit powder (DPP) and DPWF into PLA, to form bio-composites [17,18]. The specified composites attained low thermal conductivity values and high mechanical properties.

Nonetheless, the major component of DPWF, cellulose, possess a polar feature that hinders its compatibility with the polymer surface, which is non-polar [19]. Moreover, even with polar polymers, the hydrophilic feature of natural fibers leads to adhesion problems, because of the humidity intake in highly moist atmospheres. Another issue is faced by a polar polymer matrix, where the high moisture intake in humid conditions causes adhesion problems [20]. This leads to poor mechanical properties and high wettability of the composites [21]. Several studies were devoted to examining the impact of the chemical treatment of natural fibers prior to compounding them with the polymer matrix [22,23,24]. The properties of bio-composites with chemically treated filler were improved compared to composites with untreated filler of the same type [22]. Several chemical modifications for various types of natural fillers have been reported, such as peroxide treatment, sodium chlorite treatment, acrylation treatment, and acrylonitrile grafting isocyanate treatment, as well as various others [23].

One of the most common chemical treatments for natural fillers is the alkaline treatment, which is also known as mercerization. It is typically used for natural fibers prior to their incorporation into polymer matrices as a reinforcement [24]. The alkaline treatment increases the cellulose ratio and its exposure to the surface by partially removing lignin, wax, and oils covering the external surface of the fiber cell wall [25]. Bachtiar et al. [26] studied the effect of NaOH treatment with concentrations of 0.25 and 0.5 M NaOH, and different soaking times of 1, 4, and 8 h, on sugar palm fiber reinforced epoxy composites. The tensile strength increased for 0.25 M concertation and then decreased for 0.5 M concentration after 1-h immersion. When compared to the untreated filler, both 0.25 M and 0.5 M concentrations reduced the tensile strength after 4 and 8 h of immersion. It was explained that a long exposure duration or a high alkaline concentration can damage the fiber structure [26]. Alvarez et al. [27] investigated the alkaline treatment for a composite of biodegradable matrix with sisal fibers for immersion times of 25, 48, and 72 h. Furthermore, the temperature was varied between 5 °C, room temperature, and 40 °C. The best combination for increasing tensile strength was determined to be at 5 °C for 48 h [27]. Several other treatments, such as room temperature for 48 and 72 h, reduced the tensile strength relative to the untreated samples. Although it is commonly stated in the literature that alkaline treatment improves mechanical properties by removing impurities and increasing surface roughness, it was discovered that different combinations of time, concentration, and temperature conditions reduced mechanical properties by damaging the structure or causing the resulting form of fibers to be incompatible with the matrix [27].

In light of the above, it is obvious that works on the alkaline treatment of date palm fibers to reinforce biopolymers and to be used as a construction and thermal insulating construction material are lacking and need more attention. In this work, the effect of alkaline treatment on the thermal insulation and mechanical properties of DPWF-PLA composite was investigated. NaOH and KOH solutions were prepared at a concentration of 2 vol.%. DPWFs were immersed in the solution for two hours. The effect of the treatment on the composites was investigated by examining the physical, thermal, and mechanical properties. Moreover, the morphology and structure of developed composites were characterized by SEM, FTIR, and DSC analysis. This work aimed to develop bio-composites for the purpose of heat insulation by enhancing the interface between the filler and the matrix. It was expected to come up with a material with a low cost, low environmental impact, and high thermal performance.

## 2. Materials and Methods

### 2.1. Materials

PLA was obtained in pellet form from Zhejiang Zhongfu Industrial Limited in Zhejiang, China, with the following specifications: the ratio of L-lactide to D-lactide ranged from 24:1 to 32:1, with a 3.5-mm pellet diameter and a molecular weight of 2.41 × 105 g/mol. It was labeled as (4032D). PLA has a specific gravity of 1.24, semi-crystalline, and has a melting point between 155–170 °C. The date palm wood was collected from the UAE University farm in Al Foah. It contained wood waste of palm trees, obtained from different parts of the tree such as leaf, branches, and base. NaOH and KOH used in this research were in pellet form and supplied by Sigma Aldrich (St. Louis, MO, USA). Sodium Hydroxide was supplied by Sigma Aldrich, CAS: 1310-73-2, product of Czech Republic. Potassium Hydroxide was purchased from Sigma Aldrich, CAS Number: 1310-58-3, Product of Czech Republic.

### 2.2. Alkalinization and Composite Manufacturing

The wood fibers were ground using an electric grain grinder with blades (SUS304 from Yongkang Sufeng Industry and Trade Co., Ltd., Jinhua, China), and the size was reduced to 212 μm. Perforated sieve trays with an aperture of 212 μm were used to filter the size of fibers. The sieve was subject to shaking in a sieve shaker for 40 min. Afterwards, by immersing the filler in the treatment solution, an alkaline treatment was applied to the wood fiber. NaOH and KOH were dissolved in distilled water at a concentration of 2 wt.%, to form the treatment solution. The alkaline concentration was selected based on the optimum treatment conditions reported in previous studies [28]. A magnetic stirrer was used to stir the mixture at room temperature until the pellets completely dissolved. The filler particles were immersed in the treatment solution for two hours, with a ratio of 10 g of filler in 100 mL of solution (10% *w*/*v*), followed by filtration and drying in a convection oven at 95 °C for 24 h.

Composites were fabricated with 4 different filler concentrations: 10, 20, 30, and 40 wt.%. The three prepared composite types were PLA with untreated date palm wood fibers (PLA-UTDPWF), PLA with fibers treated by NaOH (PLA-NaOH), and PLA with fibers treated by KOH (PLA-KOH).

Prior to melt blending, PLA and date wood fibers, both treated and untreated, were dried at a temperature of 60 °C, until a constant weight was reached, to eliminate any moisture. Then, a double screw melt extruder (HAAKE Mini Lab II by Thermo Scientific, Dreieich, Hessen, Germany) was used to combine the PLA pellets with the filler under the following conditions: 190 °C, 140 N.m. torque, and 3-min retention time inside the extruder. Furthermore, the extruded product was placed in molds that were prepared for the various tests. The two molds employed were a square mold, which was used for the thermal conductivity test, and a cylindrical mold, which was used for water retention, compression testing, and density measurements. The square mold had dimensions of 110 × 110 × 3 mm^3^, while the cylindrical mold had a height of 25.7 mm and a diameter of 12.8 mm. The square and cylindrical molds were then placed inside a hot press machine (Carver’s AUTOFOUR/3015), to smooth the surface of the samples and shape them correctly. The hot pressing was carried out in three steps for the square (plate) mold. In the first step, a pressure of 0.50 tons was applied for 5 min and 20 s, under a temperature of 180 °C. The pressure was increased to 0.52 tons in the second stage, which lasted for 4 min, and the temperature was raised to 185 °C. The pressure was increased to 3 tons in the third stage, and the temperature was dropped to 100 °C for 3:30 min. The same pressure and temperature parameters were used for the cylindrical mold. The durations of the three stages, on the other hand, were 16 min, 10 min, and 3:30 min, respectively. The final step in the sample preparation was annealing, which took place in an oven for three hours at a temperature of 95 °C. These conditions were optimized in our previous studies [16].

### 2.3. Fourier Transform Infrared (FTIR)

Fourier transform infrared (FTIR) testing was applied to determine the functional groups present in the treated and untreated fiber, neat PLA, and the three prepared composite types. The tested filler samples were in powder form mixed with potassium bromide (KBr) salt, and were then pressed mechanically to form a disc shape. On the other hand, the neat PLA and the composites were in solid form and were cut into a disc shape. The wavelength range was taken from 600 to 4000 cm^−1^. The test was carried out using an FT/IR-4700 by JASCO. The FTIR test was applied to one sample from each category.

### 2.4. Scanning Electron Microscope (SEM)

The morphology of samples was investigated by scanning electron microscopy. The test was done using a NeoScope Scanning electron microscope provided by JOEL. Samples were coated with layer of gold prior to imaging. Moreover, images were taken with 10 Kv accelerating voltage and two different scales of 200 and 50 μm, which correspond to 100 and 500 magnifications, respectively.

### 2.5. Density (ρ)

Density measurement was carried out using a cylindrical sample. The volume was calculated after measuring sample dimensions using a Vernier caliper, and samples were weighed on a micro digital weighing balance to within 0.1 mg. The density was measured for three replicates, and the average is reported.

### 2.6. Differential Scanning Calorimeter (DSC)

A DSC test was performed to determine properties such as glass transition temperature $\left(T_{g}\right),$ melting point $\left(T_{m}\right)$, and enthalpy of melting ($∆H_{m}$). The test was performed using a differential scanning calorimeter from TA Instruments (25DSC). Crystallinity $\left(X_{c}\right)$ was found using Equation (1):

(1) $$X_{c}=\frac{∆H_{m}}{\varphi_{PLA}\cdot ∆H_{m}^{0}}$$

where $\varphi_{PLA}$ represents the PLA weight percentage in the composite, and the enthalpy of melting for pure PLA ($∆H_{m}^{0}$) is equivalent to 93.7 J/g [29]. Samples with weights ranging from 5 to 10 mg were placed inside aluminum crucibles and heated from 20 °C to 200 °C at 10 °C/min (1st heating cycle), followed by isothermal heating at 200 °C for 2 min, in order to remove the thermal history of the samples. Molten samples were cooled down to 95 °C (selected annealing temperature) at 10 °C/min and annealed isothermally for 180 min, to simulate the annealing process under a controlled atmosphere. The annealed samples were cooled to 20 °C at 10 °C/min, followed by a second heating cycle to 200 °C at 10 °C/min. One sample was tested for each category.

### 2.7. Thermal Conductivity

The thermal conductivity (*k*) was measured using a Lasercomp FOX-200 by TA Instruments, using a square-shaped specimen with dimensions of 110 × 110 × 3 mm^3^. The sample was placed between two plates with two different temperatures, to make the heat flow through the sample until equilibrium was achieved. The reported values are the average of two to three different samples.

### 2.8. Specific Heat Capacity ($C_{p}$) and Thermal Diffusivity (α)

Specific heat capacity was measured using a Differential Scanning Calorimeter Analyzer from TA Instruments (25DSC). Solid samples with weights ranging between 5 and 10 mg were placed inside aluminum crucibles. Modulated conventional test mode was applied to determine the $C_{p}$ of the samples. One sample was tested for each category.

The thermal diffusivity (α) was calculated using the measured thermal conductivity (k), the measured density (ρ), and the measured specific heat capacity ($C_{p}$), as per Equation (2):

(2) $$\alpha =\frac{k}{C_{p}\rho }$$

### 2.9. Thermogravimetry Analysis (TGA)

Thermal stability of pure PLA, untreated and treated DPWF, and composites of 20 wt.% filler content for both types of PLA-NaOH and PLA-KOH was investigated using a TGA Q500 by TA Instruments. Samples (5–10 mg) were heated from 40 to 800 °C at 20 °C/min under nitrogen atmosphere (50 mL/min). One sample was taken for each test.

### 2.10. Mechanical Properties

A compression test was performed using a universal testing machine provided by Jinan Precision Testing Equipment Co. LTD. Samples were compressed at a speed of 1 mm/s. The compression samples were fabricated using a cylindrical mold, with height of 25.7 mm and a diameter of 12.8 mm, and the reported values are the average of two to three different samples.

## 3. Results

### 3.1. FTIR

**Figure 1:**
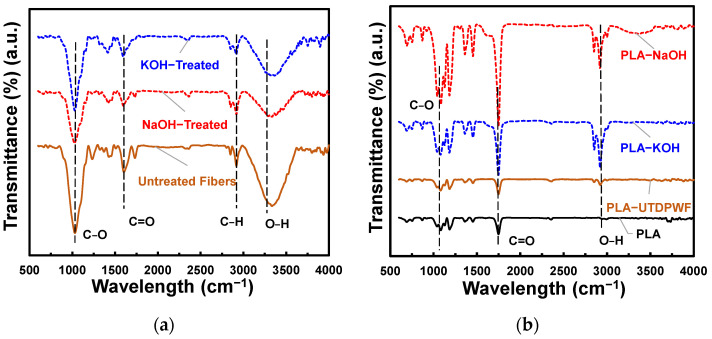
(**a**) FTIR of treated and untreated fibers; (**b**) FTIR of neat PLA, PLA-UTDPWF, PLA-NaOH, and PLA-KOH composites.

A FTIR test was performed on the fibers (treated and untreated), on pure PLA, and on composites (containing 20 wt.% of treated and untreated fibers). The FTIR results of untreated date palm fibers illustrated in Figure 1a demonstrate the presence of the C-O functional group at 1000 cm^−1^, the C=O group at roughly 1750 cm^−1^, and C-H bonding at around 3000 cm^−1^. Furthermore, the O-H group can be found at a wavelength of 3300 cm^−1^ [30]. Alkaline treatment eliminates contaminants from the surface of the filler, such as wax and dust. Moreover, it reduces lignin and hemicellulose content in the fibers. This was shown in the peak reduction at around 1250 cm^−1^, which corresponds to C-O stretching in hemicelluloses [31]. Furthermore, the peak at around 1750 cm^−1^, which represents C=O stretching in hemicellulose, was reduced for NaOH treated fibers and disappeared from KOH treated fibers. In addition, the reduction of the peak at around 1030 cm^−1^ indicates removal of the lignin content [31]. The composite, on the other hand, exhibited the functional group O-H at a wavelength of roughly 3000 cm^−1^, as seen in Figure 1b. The water absorption caused by the filler’s hydrophilic nature resulted in this peak. The cellulose ratio was higher in the alkaline treated samples because alkaline treatment eliminates the lignin and hemicellulose from the natural fiber. As a result of the hydrophilic property of cellulose, the O-H functional group was sharper in the treated composites. The carboxylic acid group (C=O) was sharper in the treated composites, possibly due to reactivity between the cellulose and the polylactic acid.

### 3.2. Microstructure of Composites (SEM)

**Figure 2:**
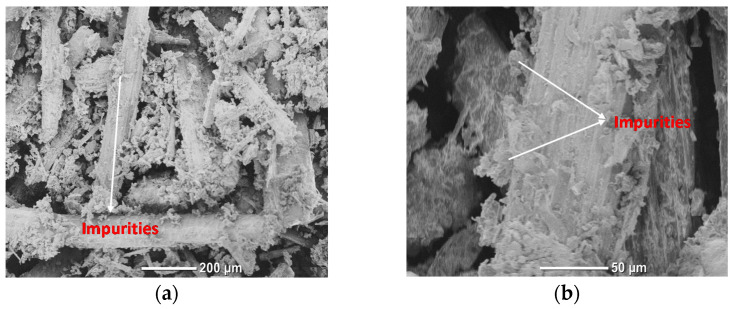

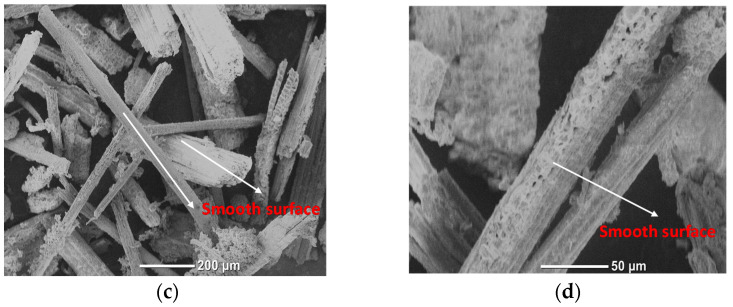
SEM of (**a**,**b**) UTDPWF, and (**c**,**d**) NaOH treated fibers.

To study the effect of the alkaline treatment on the fiber surface, SEM tests were performed on the filler particles, before and after treatment. Particles and contaminants on the surface of the untreated filler are apparent in Figure 2a,b. However, after the treatment, the surface of the fiber became notably smoother, with just a few particles remaining, as seen in Figure 2c,d. This was mostly due to the particles’ solubility in the alkaline solution, which resulted in their removal.

**Figure 3:**
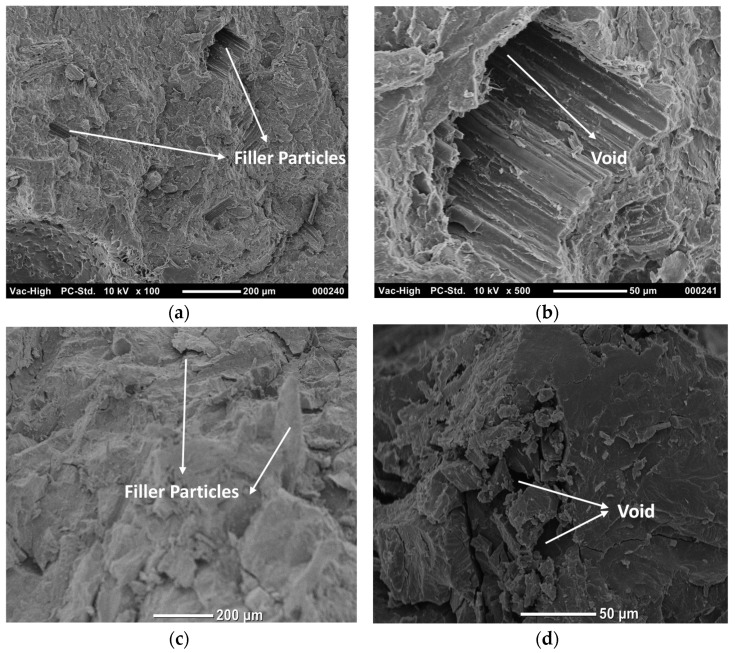
SEM of (**a**,**b**) PLA-UTDPWF, and (**c**,**d**) PLA-NaOH.

The SEM images of untreated composites in Figure 3a,b show obvious voids and hollow spaces between the filler particles and the PLA matrix. The voids occur due to poor compatibility and the rough surface of the filler particles. On the other hand, Figure 3c,d illustrate that the voids were minimized and the interface was enhanced, due to the smooth surface of the fiber after the treatment.

### 3.3. Density

**Figure 4:**
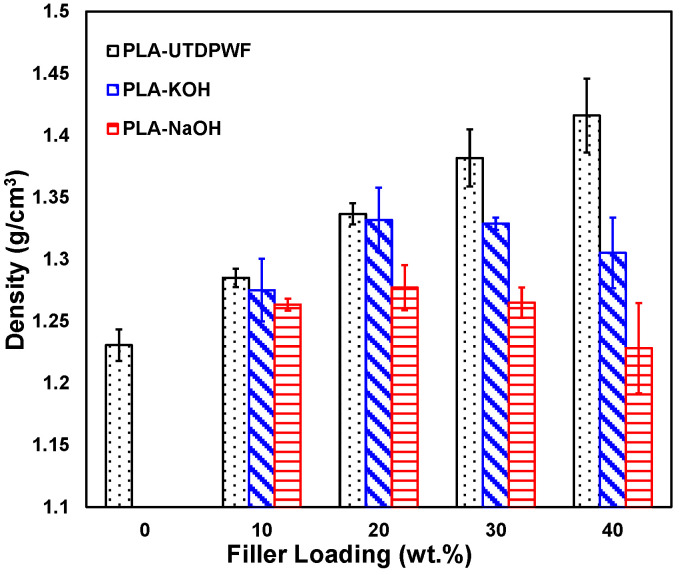
Density of PLA-UTDPWF, PLA-NaOH, and PLA-KOH composites.

The densities of PLA, date palm wood fiber, KOH treated fiber, and NaOH treated fiber were found to be 1.23, 0.52, 0.35, and 0.33 g/cm^3^, respectively. Figure 4 shows the effect of fiber treatments on the composite density at different filler contents. The density of PLA-UTDPWF composite followed a clear trend, whereby the density increased with the filler content. This may be associated with the filling of the air voids in the PLA matrix by the fibers. On the other hand, the density for the PLA-KOH and PLA-NaOH composites decreased with the 20 wt.% filler. The treatment of the filler removed lignin and other particles from the surface of the fibers, which lead to a lower density for the treated filler compared to the untreated one. PLA-NaOH composites had lower densities compared to PLA-KOH. This can be explained by the better compatibility for PLA-KOH and less air voids than the PLA-NaOH composites.

### 3.4. DSC Analysis

**Figure 5:**
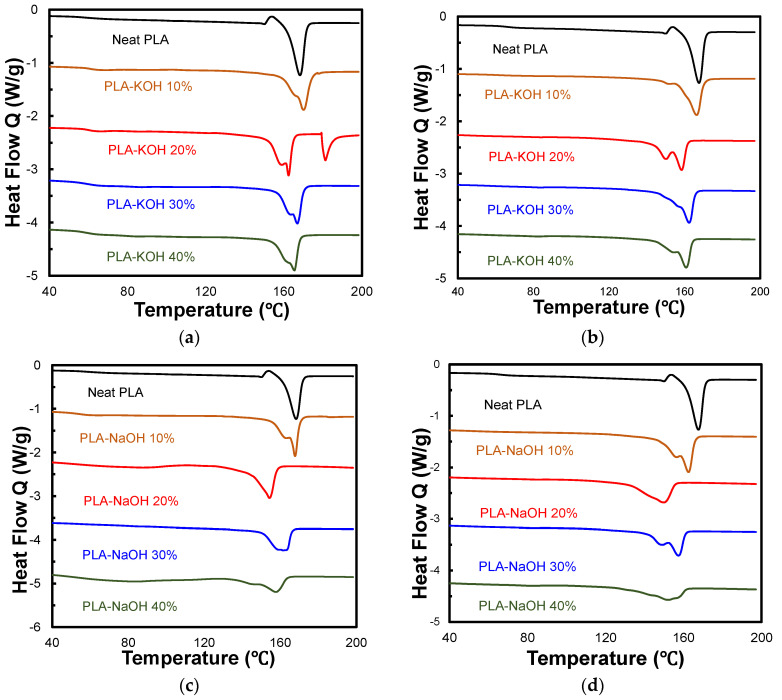
(**a**) PLA-KOH 1st heating cycle; (**b**) 2nd heating cycle; (**c**) PLA-NaOH 1st heating cycle; (**d**) 2nd heating cycle.

DSC tests were performed on all the composite types. Figure 5a,b shows the two heating cycles of PLA-KOH composites. The glass transition temperature ($T_{g}$) was determined from the first heating cycle, while the enthalpy of melting ($∆H_{m}$) and melting temperature ($T_{m}$) were determined from both cycles. The thermal properties obtained from the DSC analysis of PLA-KOH are summarized in Table 1. When the filler was added at 10 wt.%, the $T_{g}$ of the PLA-KOH composite increased significantly to 62.07 °C, compared to the $T_{g}$ of pure PLA, which was 60.72 °C. However, this then dropped for other filler contents. When the filler was first introduced at 10 wt.%, it caused anti-plasticization at low content because of filling the free volume in the polymer chain, which is opposite to the effect of plasticizer, which increases the free volume and reduces $T_{g}$. However, the increase in filler content caused a dense polymer package, which led to lower $T_{g}$ values. A similar effect was observed by Chapala, et al. [32] when making composites of poly(3-trimethylsilyltricyclononene-7) and cyclodextrines (CD) filler. They noticed that the elasticity modulus increased with introduction of small amount of CD (9 wt.%). Further increase of the CD content decreased the elasticity modulus.

On the other hand, the crystallinity of PLA-KOH composites increased with the filler content in a clear trend, starting from 47.09% and reaching 61.47%, for the 10 and 40 wt.%, respectively. For the second heating cycle, there was a slight fluctuation, but it generally had an increasing pattern, where the highest crystallinity values were achieved by the 30 and 40 wt.%. It is notable that there are two peaks for PLA-KOH 20 wt.%. They were due to two separate crystal morphologies, which can occur because of inter-lamellar and inter-spherulite, different crystalline geometries, co-crystallization and fractional crystallization, and secondary or recrystallization effects [33]. The thermal properties of the PLA-NaOH composite, presented in Table 2, showed a huge drop for the $T_{g}$ at 10 and 20 wt.% filler loadings. Nonetheless, the graphs (Figure 5c,d) do not indicate any endothermal peaks for the $T_{g}$ at 30 and 40 wt.% filler content. The results indicate a possibly damaged structure for PLA-NaOH samples at a high weight concentration, most probably due to poor compatibility. On the other hand, the crystallinity of PLA-NaOH composites was less than that of the PLA-KOH composite; it varied between 51.75 and 60.59% for the first heating cycle, and between 41.24 and 51.56% for the second heating cycles. For most of the filler loadings, the two treated types possessed a higher crystallinity than PLA-UTDPWF composites, which decreased from 58.7% for the 10 wt.%, and to 33.2% for the 40 wt.% [18]. The removal of hemicellulose and lignin may have contributed significantly to the increase in the composites’ crystallinity compared to untreated composites, as the treated filler particles were more efficient in acting as nucleating agents for PLA crystallization.

### 3.5. Thermal Conductivity

**Figure 6:**
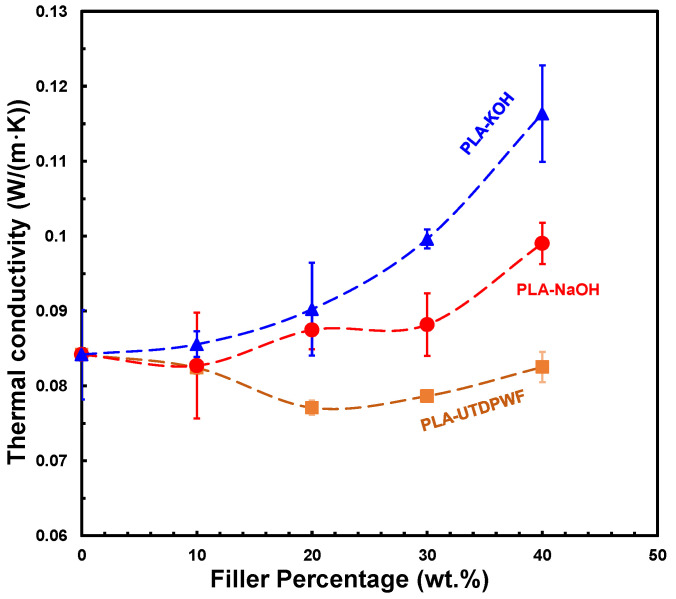
Thermal conductivity of PLA-UTDPWF, PLA-NaOH, and PLA-KOH at 25 °C.

A thermal conductivity test was conducted on the three composite types, PLA-UTDPWF, PLA-NaOH, and PLA-KOH. As can be seen in Figure 6, the thermal conductivity of PLA-UTDPWF declined with the addition of filler up to 20 wt.%, then it increased marginally. The lowest value for the thermal conductivity achieved was around 0.076$\;\frac{W}{m\cdot K}$ at 20 wt.% loading. Meanwhile, the highest value was about 0.084 $\frac{W}{m\cdot K}$, which was for the neat PLA. The thermal conductivity of pure date wood fibers was measured as 0.068 $\frac{W}{m\cdot K}$. This justifies the decline that occurred in the thermal conductivity values when the filler was incorporated. The insertion of the filler into the matrix reduced the thermal conductivity, because the thermal conductivity of the DPWF was lower than the thermal conductivity of the polymer. According to the findings of Osugi et al. [34], introducing filler creates air spaces by interrupting the polymer matrix with filler particles, which reduces the heat conductivity. Nonetheless, the increased percentage of filler particles resulted in the filling of these hollow areas. As a result of the filler’s comparatively high thermal conductivity compared to the air spaces, the thermal conductivity rose for the 30 and 40 wt.% samples. Figure 6 illustrates these outcomes.

The thermal conductivity values of both types of treated sample increased as the filler concentration increased. This was due to the involvement of cellulose inside the polymer matrix, which works as a nucleating agent that increases the crystallinity of the samples as its proportion rises. The thermal conductivity of these composite types is directly proportional to their crystallinity, which explains their growing trend in thermal conductivity [35]. The KOH-treated type achieved a higher thermal conductivity than the NaOH-treated type, indicating that it was more effective at removing wax, lignin, and other natural filler components. This is in consistent with the study of where the KOH treatment effect of Entada Mannii fibers surpassed that of NaOH and was more efficient in eliminating the lignin by alkaline cleavage and hydrolyzing the hemicellulose [36]. Furthermore, when compared to PLA-UTDPWF composites, the high moisture content, as seen in the FTIR, resulted in an increase in thermal conductivity. This is because water has a higher thermal conductivity than air voids.

The thermal conductivity of composites with treated fibers was in the range of 0.082–0.120 $\frac{W}{m\cdot K}$, where the thermal conductivity of most of the developed composites was below 0.100 $\frac{W}{m\cdot K}$, which is in the range of thermal insulators. The achieved thermal conductivity values for all three types of composites were lower than the thermal conductivity of different composites such as gypsum and date palm fibers (0.150–0.170 $\frac{W}{m\cdot K}$) [37], cork–gypsum (0.120–0.190 $\frac{W}{m\cdot K}$) [38], cement/hemp shives (0.110 $\frac{W}{m\cdot K}$) [39], and concrete/coconut (0.170 $\frac{W}{m\cdot K}$) [40].

### 3.6. Specific Heat Capacity ($C_{p}$) and Thermal Diffusivity (α)

**Figure 7:**
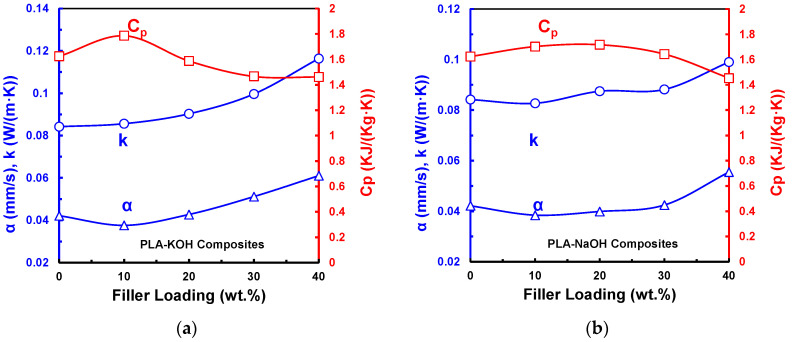
*k*, *C_p_*, and *α* for (**a**) PLA-KOH; (**b**) PLA-NaOH.

The measured $C_{p}$ of PLA-NaOH and PLA-KOH composites is presented in Figure 7. $C_{p}$ values for PLA-KOH range from 1461 to 1786 $\frac{J}{\mathrm{kg}\cdot K}$, which corresponds to filler percentages from 10 to 40 wt.%, respectively. For PLA-NaOH, this had a similar range, with slightly lower values of 1453–1703 $\frac{J}{\mathrm{kg}\cdot K}$. A different behavior was observed for a PLA-UTDPWF composite type in a previous work [18], in which the $C_{p}$ increase was directly proportional to the filler percentage, with a lower range than that of the treated types (1292 to 1487 $\frac{J}{\mathrm{kg}\cdot K}$).

The trends of $C_{p}$ with filler content are inversely proportional to the thermal conductivity. That is because $C_{p}$ for an amorphous phase is larger than the $C_{p}$ of crystalline phase [35]. The removal of hemicellulose and lignin causes a proportional increase in the crystallinity for filler loading, as was shown in the DSC analysis. Thereby, the thermal conductivity of composites increased and the $C_{p}$ decreased with the DPWF content, as illustrated in Figure 7. For thermal insulators, higher $C_{p}$ values are needed, since more energy is required to raise the temperature of the material. The two treated types outperformed various other materials in this regard, where they achieved a similar or higher $C_{p}$ than extruded polystyrene (1450–1700 $\frac{J}{\mathrm{kg}\cdot K}$), cork, (2100 $\frac{J}{\mathrm{kg}\cdot K}$), wood fibers (1900–2100 $\frac{J}{\mathrm{kg}\cdot K}$), mineralized wood fibers (1800–2100 $\frac{J}{\mathrm{kg}\cdot K}$), kenaf (1600–1700 $\frac{J}{\mathrm{kg}\cdot K}$), and jute fiber (2300 $\frac{J}{\mathrm{kg}\cdot K}$) [41].

Another critical characterization for heat insulation materials is the thermal diffusivity (α), which reflects the rate of heat transfer through the composite. Thermal diffusivity was calculated using Equation (2). Neat PLA had a diffusivity of 0.042$\;\frac{{\mathrm{mm}}^{2}\;}{s}$. As can be seen in Figure 7a, the thermal diffusivity of the PLA-KOH composite type dropped to 0.038 $\frac{{\mathrm{mm}}^{2}\;}{s}$, then continued to rise, until it reached a maximum value of 0.061 $\frac{{\mathrm{mm}}^{2}\;}{s}$ for the 40 wt.%. PLA-NaOH composites followed a similar behavior, in which the diffusivity increased in accordance with the filler percentage, ranging from 0.038 to 0.055 $\frac{{\mathrm{mm}}^{2}\;}{s}$, for the 10 wt.% and the 40 wt.%, respectively. The thermal conductivity was positively correlated with the thermal diffusivity. Therefore, the increasing pattern of the thermal conductivity values caused the thermal diffusivity to increase. The treated composites had thermal diffusivity values within the range of untreated composites, which had thermal diffusivity values ranging from 0.043 to 0.055 $\frac{{\mathrm{mm}}^{2}\;}{s}$ [18]. Moreover, they had a lower range than that of XPS (*α* = 0.544–0.638 $\frac{{\mathrm{mm}}^{2}\;}{s}$) and relatively close to that of EPS (*α* = 0.385–0.467 $\frac{{\mathrm{mm}}^{2}\;}{s}$) [42].

### 3.7. Thermogravimetric Analysis (TGA)

**Figure 8:**
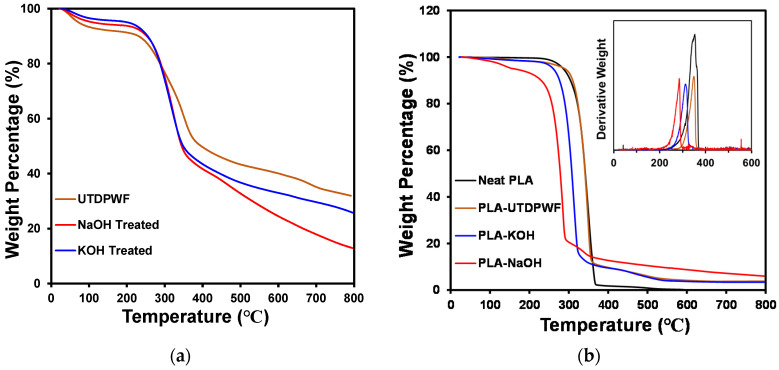
(**a**) TGA for UTDPWF, NaOH treated, and KOH treated; (**b**) TGA and DTG for PLA, PLA-UTDPWF, PLA-NaOH, and PLA-KOH composites.

Thermogravimetric analysis (TGA) was carried out for UTDPWF and treated fiber with NaOH and KOH solutions. It was also applied to the three types of composites: PLA-UTDPWF, PLA-NaOH, and PLA-KOH of 20 wt.% filler, and neat PLA. As can be observed in Figure 8a and Table 3, all three fiber types started to lose mass at around 217 °C. However, the NaOH and KOH treated fibers degrade at a faster rate than untreated date wood fiber, with NaOH achieving the lowest final residue with around only 13% at 800 °C. KOH treated fiber had a final residue of 74.0%, while UTDPWF had the highest final residue of 68.0%. For UTDPWF, the temperature at which the maximum weight loss occurred (T_max_) was around 354.32 °C. Meanwhile, the T_max_ values for KOH and NaOH were 322.78 and 327.54 °C, respectively. The treatment clearly resulted in the elimination of the particles and contaminants dissolved in the treatment solution. Thereby, the treated samples degraded at lower temperatures and had lower final residues. The NaOH treatment possibly caused surface damage to the fibers, which lead to fast and extensive degradation compared to the untreated and KOH treated fibers.

For composites, Figure 8b demonstrates that when compared to other composite types, the PLA-NaOH with 20% filler sample degraded earliest. At 226 °C, the PLA-NaOH began to degrade, and at 295 °C, it lost 80% of its weight. In accordance with the results of DSC, where no *T_g_* was observed for PLA-NaOH composites at high weight percentages, the PLA-NaOH 20 wt.% composite was the least thermally stable and degraded first. PLA-KOH composites with the same filler content decomposed at a higher temperature, starting at around 250 °C, and losing 80% of their total weight at around 333 °C. At roughly 280 °C, both pure PLA and PLA-UTDPWF began to degrade, and at around 350 °C, the PLA-UTDPWF had lost 80 percent of its total weight. The DTG shows that for neat PLA, T_max_ is at around 342 °C, which accounts for a weight loss of 43% of its initial weight. The T_max_ for PLA-UTDPWF has an almost identical value, but only 27% of the total weight was lost. On the other hand, the maximum losses for treated composites occurred at lower temperatures. The T_max_ for PLA-NaOH and PLA-KOH were 278.5 and 308.14 °C, respectively. The weight lost at the aforementioned temperatures for PLA-NaOH and PLA-KOH were 26% and 25%, accordingly. The PLA sample degraded completely, achieving a 0% final residue. Meanwhile, the three composite types had a final residue of around 5%. The treatment significantly affected the thermal stability, causing the treated samples to degrade at faster rates and at lower temperatures.

### 3.8. Compression Properties

**Figure 9:**
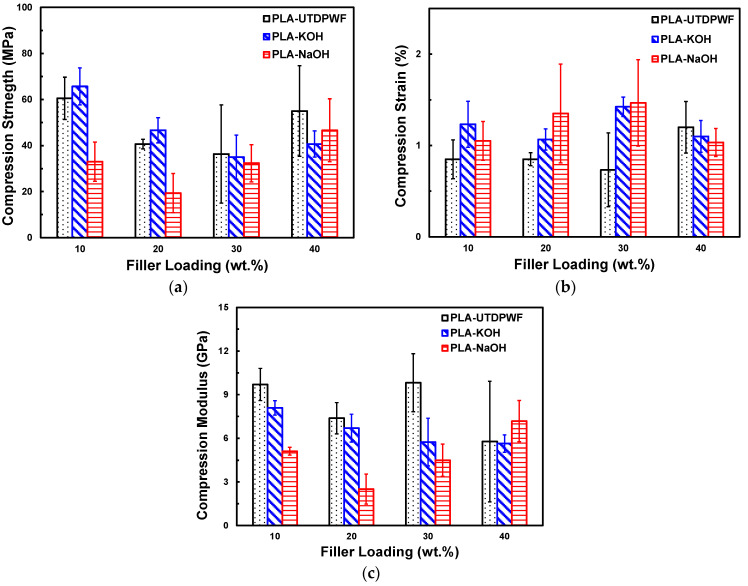
Compression Stress (**a**), Strain (**b**), and Modulus of Elasticity (**c**) for PLA-UTDPWF, PLA-KOH, and PLA-NaOH composites.

As can be seen in Figure 9a, the compression strength of the untreated composites decreased with filler content up to 30 wt.%, then it increased for the 40 wt.%. However, considering the uncertainty in the measurements, the compression strength for the 20, 30 and 40 wt.% are within a similar range of 40–55 MPa. A similar trend was observed in PLA-KOH composites, where the compression strength decreased with the filler content until 30 wt.%, then it slightly increased for a 40 wt.% loading. The PLA-KOH composite had a slightly higher compression strength than PLA-UTDPWF at low filler loadings of 10 and 20 wt.%. On the other hand, the compression strength of PLA-NaOH composite generally increased with filler content. Figure 9a shows that the PLA-KOH composite had a higher compression strength than the PLA-NaOH samples for all filler content. Moreover, the PLA-NaOH composite showed a lower compression strength than the untreated composite. This behavior and the density trend have a correlation, in which they are inversely proportional. The increase in the mechanical strength for KOH treated composites may be associated with the higher degree of crystallinity observed in PLA-KOH composites compared with PLA-NaOH. Higher crystallinity was achieved because of the better incorporation of filler, which led to stronger mechanical properties. On the other hand, Amiandamhen et al. [43] indicated that an excess concentration of alkali or suboptimal treatment condition can cause damage to the fibers and lead to weaker properties. This may possibly explain why the NaOH treated composites attained low compressive strength values in our work. Nonetheless, the prepared composites still possess higher compressive strength than the other composite materials proposed for construction, such as polystyrene-date pit [44] and cement-sheep wool fibers [45].

Regarding the compression strain, this decreased for the PLA-UTDPWF type at 20 and 30 wt.%, then it increased for the 40 wt.%. On the other hand, the PLA-KOH type had the most consistent strain among the filler percentages, ranging between 1.0 and 1.5 %. PLA-KOH had a higher strain property than PLA-NaOH and PLA-UTDPWF for the 10 and 40 wt.%, while PLA-NaOH had the highest strain before breakage at 20 and 30 wt.%, as shown in Figure 9b. The treatment enhanced the ductility of the composites, as both types of treated composites attained higher strain than PLA-UTDPWF for all filler percentages. Moreover, the modulus of elasticity is represented in Figure 9c. As illustrated, PLA-UTDPWF had the highest modulus across the majority of the filler weights. The Young’s modulus represents the resistance to deformation, by dividing stress over strain. Thereby, the results are expected, since PLA-UTDPWF had the highest stress and lowest strain of all types.

## 4. Conclusions

In this study, the impact of alkaline treatment on improving the surface interface between date palm wood fibers (DPWF) and a PLA, a biodegradable polyester, was investigated. Treatment was applied using two different alkali solutions, NaOH and KOH, on the DPWFs, prior to the incorporation of the filler into the polymeric matrix. The alkaline treatment significantly increased the crystallinity of the composites, where the crystallinity percentage of PLA-KOH and PLA-NaOH were relatively high compared to PLA-UTDPWF. The improvement in crystallinity was due to the removal of hemicellulose in natural fiber, which increased the proportion of the crystalline cellulose that acted as a nucleation agent in the composites. Moreover, the removal of the impurities and hemicellulose led to a reduction in the density for the treated composites compared to untreated ones. The treatment also increased the specific heat capacity, which is a huge advantage for thermal insulators. In addition, the compressive strength for the 10 and 20 wt.% PLA-KOH composites was higher than that of the untreated composites. Therefore, they were the most appropriate composites in this work for insulation in construction. Nonetheless, the removal of lignin and hemicellulose from the fiber exposed the surface of the cellulose to humidity and water absorption. In the case of PLA, a highly sensitive polymer to moisture, this led to a detrimental result, in terms of thermal stability. However, this problem can be solved by further treatment of the natural filler, where fibers are coated with hydrophobic layers to prevent high moisture absorption. Silane treatment/coating may be a valid suggestion for this process.

## Figures and Tables

**Table 1:** Results of DSC analysis for the PLA-KOH composite.

DWF wt.%	$T_{g}\;({}^{\circ}C)$	$T_{m}\;1\mathrm{st}\;({}^{\circ}C)$	$T_{m}\;2\mathrm{nd}\;({}^{\circ}C)$	$∆H_{m}\;1\mathrm{st}\;(J/g)$	$∆H_{m}\;2\mathrm{nd}\;(J/g)$	$X_{c}\;1\mathrm{st}\;(\%)$	$X_{c}\;2\mathrm{nd}\;(\%)$
0	60.72	168.23	168.36	37.28	34.77	39.79	37.11
10	62.07	170.12	166.96	39.71	38.77	47.09	45.97
20	60.76	162.36	159.11	40.29	32.36	53.75	43.16
30	58.52	166.78	162.98	38.79	33.77	59.14	51.48
40	58.55	165.33	161.53	34.56	28.54	61.47	50.77

**Table 2:** Results of DSC analysis for the PLA-NaOH composite.

DWF wt.%	$T_{g}\;({}^{\circ}C)$	$T_{m}\;1\mathrm{st}\;({}^{\circ}C)$	$T_{m}\;2\mathrm{nd}\;({}^{\circ}C)$	$∆H_{m}\;1\mathrm{st}\;(J/g)$	$∆H_{m}\;2\mathrm{nd}\;(J/g)$	$X_{c}\;1\mathrm{st}\;(\%)$	$X_{c}\;2\mathrm{nd}\;(\%)$
0	60.72	168.23	168.36	37.28	34.77	39.79	37.11
10	54.06	167.37	162.97	43.64	43.48	51.75	51.56
20	54.76	154.07	150.48	45.42	32.52	60.59	43.38
30		161.56	158.07	37.84	30.24	57.69	46.10
40		157.82	153.09	29.16	23.19	51.86	41.24

**Table 3:** Decomposition temperature and overall change in weight of the composites and fibers.

Composites/Filler	T5 (°C) *	T50 (°C) **	T_max_ (°C)	Overall Weight Loss (%)
Neat PLA	285.78	342.0	353.76	100
PLA-UTDPWF	290	341.41	351.0	96.25
PLA-KOH	260.49	308.14	314.4	96.58
PLA-NaOH	159.0	278.47	286.4	94.0
UTDPWF	72.27	396.16	354.32	68.0
KOH Treated	202.27	348.7	322.78	74.0
NaOH Treated	110.77	344.61	327.54	87.0

* Temperature at which 5% weight loss occurs. ** Temperature at which 50% weight loss occurs.

## Data Availability

The data presented in this study are available on request from the corresponding author.
